# Outcomes and Cost-Effectiveness of Two Nicotine Replacement Treatment Delivery Models for a Tobacco Quitline

**DOI:** 10.3390/ijerph8051547

**Published:** 2011-05-13

**Authors:** Jessie E. Saul, Rebecca Lien, Barbara Schillo, Annette Kavanaugh, Ann Wendling, Michael Luxenberg, Lija Greenseid, Lawrence C. An

**Affiliations:** 1 North American Research & Analysis, Inc., 1016 11th Ave. NE, Faribault, MN 55021, USA; 2 Professional Data Analysts, Inc., Minneapolis, MN 55414, USA; E-Mails: blien@pdastats.com (R.L.); kavan003@hotmail.com (A.K.); michael@pdastats.com (M.L.); lija@pdastats.com (L.G.); 3 ClearWay Minnesota^SM^, Minneapolis, MN 55425, USA; E-Mails: bschillo@clearwaymn.org (B.S.); awendl9402@aol.com (A.W.); 4 General Medicine, University of Michigan, Ann Arbor, MI 48109, USA; E-Mail: lcan@umich.edu

**Keywords:** tobacco cessation, program evaluation, cost effectiveness, cessation medications, NRT, nicotine replacement therapy

## Abstract

Many tobacco cessation quitlines provide nicotine replacement therapy (NRT) in the U.S. but consensus is lacking regarding the best shipping protocol or NRT amounts. We evaluated the impact of the Minnesota QUITPLAN^®^ Helpline’s shift from distributing NRT using a single eight-week shipment to a two-shipment protocol. For this observational study, the eight week single-shipment cohort (n = 247) received eight weeks of NRT (patches or gum) at once, while the split-shipment cohort (n = 160) received five weeks of NRT (n = 94), followed by an additional three weeks of NRT if callers continued with counseling (n = 66). Patient satisfaction, retention, quit rates, and cost associated with the three groups were compared. A higher proportion of those receiving eight weeks of NRT, whether in one or two shipments, reported that the helpline was “very helpful” (77.2% of the single-shipment group; 81.1% of the two-shipment group) than those receiving five weeks of NRT (57.8% of the one-shipment group) (p = 0.004). Callers in the eight week two-shipment group completed significantly more calls (3.0) than callers in the five week one-shipment group (2.4) or eight week single-shipment group (1.7) (p < 0.001). Using both responder and intent-to-treat calculations, there were no significant differences in 30-day point prevalence abstinence at seven months among the three protocol groups even when controlling for demographic and tobacco use characteristics, and treatment group protocol. The mean cost per caller was greater for the single-shipment phase than the split-shipment phase ($350 *vs.* $326) due to the savings associated with not sending a second shipment to some participants. Assuming no difference in abstinence rates resulting from the protocol change, cost-per-quit was lowest for the five week one-shipment group ($1,155), and lower for the combined split-shipment cohort ($1,242) than for the single-shipment cohort ($1,350). Results of this evaluation indicate that while satisfaction rates increase among those receiving more counseling and NRT, quit rates do not, even when controlling for demographic and tobacco use characteristics.

## 1. Introduction

Proactive telephone counseling is an effective treatment for helping people quit smoking [1,2] and has been recommended by the 2008 Clinical Practice Guideline (2008 Guideline) [3]. Since the establishment of the first telephone-based cessation service by the U.S. Cancer Information Service in the early 1980s [4], telephone quitlines have been established in all 50 of the United States, Washington, DC, and Puerto Rico, as well as all states and provinces in Canada and Australia, in most European countries, and in several other parts of the world [4,5]. Additionally, a single national access number (1-800-QUIT-NOW) has been established in the United States [5].

The widespread adoption of telephone quitlines has been accompanied by a growing literature on their effectiveness [6–9]. Additionally, the combination of telephone quitline counseling with other modes of treatment, particularly various forms of nicotine replacement therapy (NRT), has proven to be even more effective than either form of treatment alone [3,10,11]. Drawing similar conclusions to the 2008 Guideline the authors of the Cochrane review on nicotine replacement therapy for smoking cessation stated that “the absolute increase in success rates attributable to the use of NRT will be larger when the baseline chance of success is already raised by the provision of intensive behavioural support” [12].

Currently 43 state quitlines offer some type of free cessation medications to at least some of their callers [13]. There is little published research, however, regarding the “real world” impact of delivering NRT on quit outcomes. New York State offered varying amounts and types of NRT in conjunction with its Smokers’ Quitline in 2003. Quit rates measured at four months varied in relationship to the supply of NRT sent to participants, but were all higher than among smokers not sent NRT (21–35% *vs.* 12%) [14]. Smokers in New York City who were sent a six-week supply of nicotine patches had a 12-month 7-day point prevalence abstinence rate 1.78 times higher than among a comparable group of smokers using the quitline who did not receive them [15]. We have reported elsewhere that 30-day abstinence for the Minnesota QUITPLAN^®^ Helpline measured at six months increased from 10.0% to 18.2% following the addition of NRT to the quitline [16].

In addition to greater efficacy, provision of free medications appears to motivate tobacco users to call quitlines, resulting in large increases in call volume [14–23]. It also appears to be associated with an increase in the number of contacts that quitlines have with tobacco users, resulting in opportunities for additional counseling [20,23,24]. Other studies have demonstrated that providing NRT results in a greater number of individuals both progressing to the counseling phase and adhering to treatment [23]. Given the link between the number of calls and effectiveness of counseling [3], providing medication appears to have a synergistic effect on counseling effectiveness.

For quitlines considering the provision of NRT, questions remain as to the most effective mechanisms and timing of dose delivery. In the 2007 Cochrane review on NRT for smoking cessation, the authors concluded that there was no additional benefit from providing more than an 8-week supply of nicotine patch [12]. A more recent article found no difference in quit rates between smokers receiving 4-, 6-, and 8-week supplies of nicotine patches through a quitline service at 12 months [25]. Smaller trials similarly found no difference in quit rates between shorter and longer courses of nicotine patch [26,27] although they were not quitline trials.

While there is growing evidence to support the effectiveness of providing NRT as part of quitline services, important questions remain as to how NRT should be provided. One basic question is whether the entire course of NRT should be provided in one shipment or split into two or more shipments. Providing one single shipment is straight forward and easy to implement, and assures there will be no interruption in the supply of NRT for an individual. Providing split shipments requires more administrative effort and costs (e.g., tracking, mailing). It does, however, offer some potential advantages in terms of cost savings from not distributing additional NRT to individuals who have relapsed to smoking. A split shipment protocol may also encourage callers to participate in additional counseling sessions if continued counseling is required to receive a second shipment of NRT.

To our knowledge, there are no prior reports comparing these two distribution strategies. We address this gap by reporting on the experience of the Minnesota QUITPLAN Helpline using a single *vs.* two shipment protocol for NRT. Patient satisfaction, retention, quit rates, and cost per quit associated with these approaches to distributing NRT are examined. This information will be of practical importance to employers, health plans, and local, state, and national agencies that seek to improve delivery of tobacco treatment services and reduce the burden of tobacco-related disease.

### Setting

ClearWay Minnesota^SM^ was created in 1998 with 3 percent of the state’s tobacco settlement and is an independent, nonprofit organization. Its mission is to enhance life for all Minnesotans by reducing tobacco use and exposure to secondhand smoke through research, action and collaboration. In September 2001, ClearWay Minnesota began providing proactive telephone counseling services to under- and un-insured Minnesotans (ClearWay Minnesota defines “under-insured” as callers to the quitline who have health insurance, but who do not have insurance coverage for *either* telephone counseling *or* nicotine replacement therapy). Together in partnership with seven major health plans in Minnesota, the QUITPLAN Helpline provides statewide access to telephone counseling. Callers to the QUITPLAN Helpline in 2001 were served by Free & Clear, Inc.

In September 2002, the Minnesota QUITPLAN Helpline began providing eight weeks of nicotine replacement therapy (patches or gum) at no cost or co-pay to eligible helpline callers, which impacted both call volumes and quit outcomes [16,28]. Callers were required to sign up for the multi-session counseling program in order to receive one eight-week shipment of NRT. Direct shipment of NRT was elected over vouchers to eliminate an additional barrier to the use of NRT for tobacco users wanting to quit.

On August 1, 2003, the NRT dosing protocol shifted from one eight-week shipment to two shipments—first a five-week supply followed later by a three-week supply. The second shipment was sent only if callers completed another counseling call shortly before they were scheduled to run out of NRT, and if certain additional conditions were met. Conditions included that the caller was still in an active quit attempt, reported no new medical contraindications, and had experienced no adverse effects from the NRT product to be provided. The decision was made to split the shipment of NRT to provide flexibility in dosing in the case of discontinuation or problems with the first form of NRT, to encourage continuing contact with the tobacco counselor, and to avoid possible product wastage and the associated increased cost. Addressing the issues of product wastage and the potential cost savings associated with eliminating it were deemed critical given that earlier surveys showed that the average duration of NRT use was less than the full eight weeks (unpublished ClearWay Minnesota evaluation data).

## 2. Research Methods and Outcome Measures

ClearWay Minnesota contracted with Professional Data Analysts, Inc. (PDA) to evaluate the QUITPLAN Helpline. Data sources included program registration information (demographic characteristics, tobacco use and quitting history, and readiness to quit), helpline administrative records (dosing and mailing dates for each shipment of NRT, contraindications for NRT, number of counseling calls, costs), and phone surveys administered six months after registration (satisfaction, quit attempts, self-reported medication use, cessation outcomes).

All callers to the helpline were eligible to be part of the evaluation if they: (1) requested counseling services for themselves and (2) were 18 or older at registration. Two study periods were selected: one during the single NRT shipment period (May–July 2003) and one during the split NRT shipment period (July–September 2004). The total number of callers in each study period was 353 and 301, respectively. Those excluded from the evaluation are summarized in Table 1.

All callers meeting these criteria during the two time periods for the study were included in the evaluation: the single NRT shipment period (n = 247) and the split NRT shipment period (n = 160). Cohort members were not randomized to either study condition. Of the 160 participants in the split-shipment cohort, 94 received only one five-week shipment of NRT, while 66 received the first five-week shipment plus a second three-week shipment for a total of eight weeks of NRT shipped. For the purposes of this analysis, the three NRT protocol groups will be compared (“eight week single-shipment group”, “five week one-shipment group”, and “eight week two-shipment group”).

For follow-up evaluation surveys six months after registration, up to seven call attempts were made by a survey subcontractor, and up to an additional 25 call attempts were made by the evaluator if the participant was unable to be reached either because seven unsuccessful attempts had been made or because the telephone number was unusable and a reverse address lookup phone number was found.

Measures were designed to assess the impact of a split-dosing NRT protocol on caller satisfaction, retention, quit rates and cost per quit. Satisfaction with the program was measured by asking two questions: “How helpful was the QUITPLAN Helpline as a whole?” with response options of “not helpful at all/a little helpful/somewhat helpful/very helpful” and “Would you recommend the helpline to a friend who is trying to quit?” with response options of “Yes/Maybe/No”. The primary measure of retention was the average number of counseling calls completed by speaking to a “live” counselor. A secondary measure of retention was the average number of minutes of counseling. The primary cessation outcome was self-reported abstinence from all tobacco products for 30 days or longer at the time of the six-month follow-up. Abstinence rates are presented two ways: (1) for survey respondents only and (2) respondents plus non-respondents by assuming all non-respondents have not quit (intent-to-treat). Cost-per-quit within each NRT dosing category was calculated by multiplying the number of people in each category by the average cost per caller for each category, and dividing by the number of people reporting being quit for the past 30 days at 6-months within each category. Cost-per-quit estimates were calculated using intent-to-treat 30-day abstinence rates for all three groups separately, as well as the overall quit rate for all participants combined.

Costs for both counseling and NRT varied over the time period in question. While actual costs are available for both periods, the pricing structure changed over time, making direct comparison of actual costs difficult. To remove the potential effect of changes in pricing over time, standardized costs were used to isolate and examine the effects of splitting the NRT shipment. Actual call volume and callers served numbers were used for all calculations for both time periods. All costs are from the perspective of the organization funding (incurring costs) counseling and NRT (ClearWay Minnesota), and are presented in 2004 dollars. For both the single-NRT shipment period and the split-NRT shipment period, the estimated cost of $175 per participant was used for counseling alone. For the eight week single-shipment period, an additional $175 was estimated as the cost of providing NRT. (Note: this does not provide an estimate of the differences in cost between patches and gum, but a single equilibrated cost estimate for both types of NRT). For the split-shipment NRT period, the first 5-week shipment was estimated to cost $120, and the second 3-week shipment was estimated to cost $75. The total cost for NRT was greater during the split-shipment phase due to additional administrative and shipping costs.

Analysis for this study was performed by PDA using SPSS 15.0 and 18.0. Comparison of caller characteristics for the eight week single-shipment cohort, the five week one-shipment group and the eight week two-shipment group during the split-shipment period was performed using chi-square for categorical variables and t-test or non-parametric tests for continuous variables. Caller retention comparisons were conducted using ANOVAs. Unadjusted comparison of abstinence outcomes was performed using chi-square tests. Assessment of the three protocol treatment groups on the same outcomes also was done after controlling for demographic and tobacco use history variables at intake using a logistic regression modeling approach. This study was reviewed by the University of Minnesota’s Institutional Review Board and determined to be exempt under federal guidelines 45 CFR 45.101 (b) for existing data.

## 3. Results

### 3.1. Survey Response

The survey response rates among the three protocol groups were not statistically significant. The response rates were 74.5% for the eight week single-shipment cohort, 68.1% for the five week one-shipment group, and 80.3% for the eight week two-shipment group (p = 0.21).

### 3.2. Demographic and Clinical Characteristics

The key demographic and clinical characteristics for the eight week single-shipment group, the five week one-shipment group, and the eight week two-shipment group are displayed in Table 2. There were no significant differences in the proportion of study participants between the three groups by gender, marital status, employment status, ethnicity (white/non-white), educational level, readiness to quit, time to first cigarette, or quit attempts in the prior year. The five week single-shipment group differed from those who received the full eight week supply of NRT (either in one or two shipments) in two ways. There were more 18–24 year olds in the five week one-shipment group (20.2%) compared to the eight week one-shipment (10.1%) and eight week two-shipment group (3.0%) (p < 0.01). There was a higher proportion of participants in the 7-county metro region (greater Minneapolis/St. Paul metro area) in the five week one-shipment group (68.1%) than in either the eight week single-shipment group (53.4%) or the eight week two-shipment group (54.5%) (p = 0.05). In the eight week single-shipment group there were fewer uninsured callers than in the one- and two-shipment groups (28.5% *vs.* 45.5% and 41.5% respectively, p < 0.01), and fewer heavy smokers (17.8% *vs.* 33.0% and 24.2% p = 0.01), than the one- and two-shipment groups.

### 3.3. Use of NRT

Among survey respondents only, while there was a significant difference in the average duration of use of NRT overall between the three groups (p = 0.007), post-hoc tests showed that participants in the two-shipment group used NRT significantly longer (48.8 days) than those in the single shipment group (33.5 days) or the one-shipment group (28.9 days).

### 3.4. Caller Satisfaction

Satisfaction was measured by participants’ response to the question “how helpful was the QUITPLAN Helpline as a whole?” While nearly three-quarters (73.8%) of all respondents indicated the helpline was “very helpful,” responses differed significantly by NRT protocol group. A higher proportion of those receiving eight weeks of NRT reported that the helpline was “very helpful” (77.2% of the single-shipment group; 81.1% of the two-shipment group) than those receiving five weeks of NRT (57.8% of the one-shipment group) (p = 0.004). There was no difference in the proportion reporting the helpline was “very helpful” between the two eight-week groups (single or two-shipment). A logistic regression model controlling for demographics and tobacco use history, confirmed these results. The following variables were permitted entry into the model in stepwise fashion: Block 1 included demographic characteristics [gender, age (18–24/25+), region (metro/non-metro), race (white *vs.* other), education level (3 levels), and insurance status (y/n)]; Block 2 included baseline (intake) tobacco use characteristics [cigarettes smoked per day(<25/25+), time to first cigarette (<30 mins/31+), number of quit attempts in the past year(0/1+)]; Block 3 included the number of calls completed live(0,1/2,3,4+); and Block 4 included the treatment protocol groups (eight-week single-shipment, five-week one-shipment, or eight week two-shipments). The only variable entering the hierarchical model was the protocol group.

### 3.5. Caller Retention

Callers in the eight week two-shipment group completed significantly more calls (3.0) than callers in the five week one-shipment group (2.4) or eight week single-shipment group (1.7) (p < 0.001). The difference between the number of calls completed for the eight week and five week single-shipment groups was not significant. In addition, callers in the eight-week single-shipment and five-week one-shipment groups had significantly fewer average minutes of counseling than those in the eight week two-shipment group (mean of 45.8 minutes for the eight week single-shipment group, 42.8 minutes for the five week one-shipment group, and 66.6 minutes for the eight week two-shipment group, ANOVA, p < 0.001). The difference in the mean number of minutes of counseling completed for those in the eight week single-shipment group and those in the five week one-shipment group was not significant.

### 3.6. Cessation Outcomes

Using both responder and intent-to-treat calculations, there were no significant differences for the primary cessation outcome variable among the three protocol groups. Assuming all non-responders were still smoking at the time of follow-up (ITT), 30-day point prevalence abstinence rates were 28.3% for the eight week single-shipment group, 18.1% for the five week one-shipment group and 28.8% for the five plus three two-shipment group (p = 0.134). Among responders only, 30-day point prevalence abstinence rates were 38.0% for the eight week single-shipment group, 26.6% for the five week one-shipment group and 35.8% for the eight week two-shipment group (p = 0.252).

Because there were some demographic and tobacco use history differences at intake among the three groups as shown in Table 2, a logistic regression was run for all responders to control for potential confounding factors on 30-day abstinence rates. Variables were permitted entry into the model in stepwise fashion using the same blocks as the satisafaction outcomes model above. No variables entered the model; that is, after controlling for all demographic and tobacco use characteristics, as well as levels of treatment, the NRT shipment protocol groups did not have different 30-day quit rates. When all cases were included in the model (both responders and non-responders), assuming that all non-responders were still smoking at six months, two variables entered the model—education level, and number of live counseling calls—but NRT protocol group did not. The results of the logistic regression analysis allow us to conclude that the lack of statistically significant differences in quit rates among protocol groups is not an artifact of differences of the composition of the protocol group participants themselves, or of the level of treatment they received.

### 3.7. Cost per Caller and Cost per Quit

The cost per caller for the eight week single-shipment phase was $350, and the cost per caller for the split-shipment phase was $326. Despite the increased total cost for the full eight weeks of NRT in the split-shipment phase ($195 *vs.* $175) due to greater administrative and shipping costs, the mean cost per caller was lower during the split-shipment phase due to fewer people receiving the second shipment of NRT.

Cost-per-quit estimates were calculated using intent-to-treat 30-day abstinence rates for all protocol groups. Results are presented in Table 3.

Using both the intent-to-treat analysis and responder analysis we find that the cost-per-quit was more expensive for the five-week one-shipment group ($1,109–$1,631 per quit) than for either group who received eight weeks of NRT. Combining the five week one-shipment and eight week two-shipment groups together, the total cost-per-quit for the split-shipment cohort ranged from $1,058–$1,449, which was more than the total cost-per-quit for the single shipment eight-week cohort ($921–$1,235).

The analysis presented in Table 3 used actual quit rates observed among the study participants. However, given the results of the logistic regression for 30-day point prevalence abstinence at 6 months presented above showing no significant difference in 30-day quit rates between the three groups, the cost-per-quit calculation was repeated using a single overall quit rate for all study participants (grouping all participants and assuming all non-responders were still smoking) in order to avoid spurious differences between protocol groups artificially influencing the cost analysis.

Using the intent-to-treat overall quit rate of 26.0% and the standardized cost for each NRT protocol group as described above, the cost per quit for the eight week single-shipment group ($1,350) was more than the cost per quit for the five week one-shipment group ($1,155), but less than the five plus three two-shipment group ($1,436). Combining the five week and eight week groups together, the total cost-per-quit for the split-shipment cohort was $1,242, which was less than the cost-per-quit of the single-shipment cohort ($1,350).

## 4. Discussion

### 4.1. Discussion of Results

This study found splitting the full eight-week dose of NRT for tobacco cessation quitline callers into two shipments reduced the cost of providing medications per caller. Number of weeks of NRT used did not differ between the eight week single-shipment group and the five week one-shipment group, but increased significantly with the eight week two-shipment group. Satisfaction was high for both the single and two-shipment groups receiving eight weeks of NRT, but significantly lower for those receiving only one shipment (five weeks) of NRT. There was no difference in the number of calls completed or the number of minutes of counseling for those in the eight week single- and five week one-shipment groups, but those in the eight week two-shipment group completed significantly more calls and minutes of counseling. Yet despite the differences in satisfaction, amount of NRT used, number of calls completed, and number of minutes of counseling, quit rates did not differ significantly between the three groups, even when controlling for demographic and tobacco-use characteristics and NRT protocol group assignment.

Cost-per-quit was highest for the five week one-shipment group using observed quit rates. However, assuming no differences in quit rates result from the change in protocol, the cost-per-quit was in fact lowest for the five week one-shipment group, and lower for the combined split-shipment cohort than for the single-shipment cohort. Participants who completed more calls received more NRT and more total minutes of counseling. They also reported higher levels of satisfaction. Splitting the shipment of NRT translated to lower overall costs for the quitline per caller. Assuming no differences in quit rates result from the change in protocol, it also translates to lower overall costs for the quitline per quitter.

It is not clear why the more intensive treatment did not result in higher quit rates. The results shown here support those of other studies that failed to find differences in quit rates between groups receiving varying amounts of NRT [12,25–27]. It may be that quitlines with limited resources could achieve satisfactorily high quit rates while offering a smaller amount of NRT to callers, however further research is warranted to determine the optimum amount and distribution protocol for NRT for quitlines, including the option of receiving NRT only with no counseling. Studies are also needed that compare direct mailing of NRT (as with the present study) to alternative methods of providing NRT such as vouchers. While vouchers could potentially save quitlines a significant amount of money due to a proportion of them never being used by smokers, it is unclear what the cost in terms of lower quit rates might be because fewer callers would be using NRT.

### 4.2. Limitations

The observational nature of this study produced several limitations. No random assignment occurred to the single-shipment and split-shipment conditions, nor between the one- and two-shipment conditions within the split-shipment cohort. In fact, inclusion in the eight week two-shipment group was dependent on the number of calls completed. In addition, the dates of enrollment and follow-up for the two studies did not align exactly, making it impossible to control for the potential impact of seasonality. Although the sample sizes are reasonably large (cohort 1:247, cohort 2: 94 + 66 = 160) to allow us to detect large differences, smaller differences would be harder to detect. An analysis was performed to determine what the difference in 30 day (intent-to-treat) quit rates would need to be in order to reject the null of no difference between the two cohorts 80% of the time; this was estimated to be 12.4%. If the population difference is less than this, then statistical comparisons made between samples drawn of the same size would be less likely (less than 80% of the time) to reject the hypothesis of no difference. While no major state-wide policy changes went into effect during the study periods, such as cigarette tax increases or smoke-free policies, there may have been other changes in the environment that contributed to some of the results reported, such as local policy initiatives.

## 5. Conclusions

This study adds an NRT delivery model for quitlines to consider when providing NRT to callers. Results of this evaluation indicate that while satisfaction rates increase among those receiving more counseling and NRT, this study did not find evidence that quit rates increased, even when controlling for demographic and tobacco use characteristics. Assuming no change in quit rate with the change in NRT dosing protocol, cost per caller and cost per quit are lower using a split-shipment delivery model. It will be important to replicate this study with a more fully powered sample to better understand the cost implications of splitting an eight-week shipment of NRT. While this study focused on splitting an eight-week shipment, additional research into how best to provide NRT to quitline callers to yield optimal cessation outcomes is needed. Absent such research, quitlines must weigh their unique goals and available resources to determine whether and how to deliver NRT to quitline callers in the most efficacious manner.

## Figures and Tables

**Table 1.** Inclusion and Exclusion criteria by NRT protocol group.

**Inclusion Criteria**	**Single Shipment Protocol Period (May–July 2003)**		**Split Shipment Protocol Period (July–September 2004)**	

	**Excluded**	**Included**	**Excluded**	**Included**

Total calls		353		301
Calling for self	10	343	2	299
Served by the helpline	14	329	0	299
Consented to inclusion data in public reporting	3	326	5	294
Still smoking at registration	13	313	88	206
Dosed for NRT	66	247	46	160
Total included in study		247		160 (94 received 5 weeks of NRT; 66 received 5 + 3 weeks of NRT)

NRT = Nicotine Replacement Therapy.

**Table 2.** Baseline characteristics of callers by NRT protocol group.

**Variable**	**Single shipment cohort 8 weeks of NRT (N = 247)**		**Split shipment cohort 5 weeks of NRT (N = 94)**		**Split shipment cohort 5 + 3 weeks of NRT (N = 66)**		**p**

	N	%	N	%	N	%	

Responder to 6 month survey	184	74.5%	64	68.1%	53	80.3%	0.21
Gender—female	134	54.7%	50	53.2%	39	60.0%	0.68

**Age**							**<0.01**

18–24	**25**	**10.1%**	**19**	**20.2%**	**2**	**3.0%**	
25+	**222**	**89.9%**	**75**	**79.8%**	**64**	**97.0%**	

**Metro (7-county metro)**	**132**	**53.4%**	**64**	**68.1%**	**36**	**54.5%**	**0.05**

Married	46 (of 92)	50.0%	26	38.2%	28	50.9%	0.25
Employed	60 (of 92)	65.2%	45	66.2%	32	58.2%	0.61
Ethnicity/race (non-White)	39 (of 244)	16.0%	10 (of 92)	10.9%	8 (of 63)	12.7%	0.45
Education	N = 239		N = 92		N = 64		0.59
High school or less	107	44.8%	48	52.2%	26	40.6%	
Some college	90	37.7%	28	30.4%	27	42.2%	
College grad/post-grad	42	17.6%	16	17.4%	11	17.2%	

**Health insurance status—Uninsured**	**70 (of 246)**	**28.5%**	**40 (of 88)**	**45.5%**	**27 (of 65)**	**41.5%**	**<0.01**

Readiness to quit (ready to quit in the next 30 days)	239	96.8%	86	91.5%	63	95.5%	0.12

**Cigarettes per day**	**N = 247**		**N = 94**		**N = 66**		**0.01**

Light-Mod. (<25 cigs/day)	203	82.2%	63	67.0%	50	75.8%	
Heavy (25+ cigs/day)	44	17.8%	31	33.0%	16	24.2%	

Time to first cigarette	N = 245		N = 84		N = 60		0.06

<30 min	182	74.3%	71	84.5%	51	85.0%	
31 or more min	63	25.7%	13	15.5%	9	15.0%	

Quit attempts prior year	N = 247		N = 93		N = 66		0.36

0	14	5.7%	7	7.5%	7	10.6%	
1 or more	233	94.3%	86	92.5%	59	89.4%	

All p values calculated by chi-square test; Mod = Moderate; NRT = Nicotine Replacement Therapy; Bold text indicates statistically significant findings (p < 0.05).

**Table 3.** Cost per quit using ITT 30-day abstinence rates.

	**Dosing group**	**N (entire sample)**	**% of sample**	**Cost per caller**	**Total Cost**	**30-day PPA (ITT)**	**Cost per quit (ITT)**	**30-day PPA (RR)**	**Cost per quit (RR)**
**Single-shipment**	**Eight-weeks single-shipment**	247	100%	$350	$86,450	28.3%	**$1,235**	38.0%	**$921**
**Split-shipment**	Five-weeks One-shipment	94	58.8%	$295	$27,730	18.1%	$1,631	26.6%	$1,109
	Five-plus-three weeks Two-shipments	66	41.3%	$370	$24,420	28.8%	$1,285	35.8%	$911
	**Split-shipment combined**	160	100%	$326	$52,150	22.5%	**$1,449^a^**	30.8%	**$1,058^a^**

a Weighted average cost per quit calculated by multiplying the average cost per quit by the % of sample, and adding those results together; PPA = point prevalence abstinence; ITT = intent-to-treat; RR = responder rate.
